# Comparative screening of endosymbiotic bacteria associated with the asexual and sexual lineages of the termite *Glyptotermes nakajimai*

**DOI:** 10.1080/19420889.2019.1592418

**Published:** 2019-03-20

**Authors:** Toshihisa Yashiro, Nathan Lo

**Affiliations:** School of Life and Environmental Sciences, University of Sydney, Sydney, NSW 2006, Australia

**Keywords:** All-female asexual societies, thelytokous parthenogenesis, reproductive parasites, social insects

## Abstract

Males provide opportunities both for sexual reproduction and for sex-based phenotypic differences within animal societies. In termites, the ubiquitous presence of both male and female workers and soldiers indicate that males play a critical role in colonies of these insects. However, we have recently reported all-female asexual societies in a lineage of the termite *Glyptotermes nakajimai* – a dramatic transition from mixed-sex to all-female asexual societies. It is known that female-producing parthenogenesis in insects can be induced by maternally inherited endosymbiotic bacteria, such as *Wolbachia, Cardinium*, and *Rickettsia*. Here, we screen for the presence of endosymbiotic bacteria in the asexual and sexual lineages of *G. nakajimai*. Our bacterial screening of the asexual lineage did not reveal any likely causal agents for parthenogenetic reproduction, whereas screening of the sexual lineage resulted in *Wolbachia* being detected. Our findings suggest that the asexuality in *G. nakajimai* is likely to be maintained without manipulation by endosymbiotic bacteria.

## Introduction

Both males and females of many animals invariably participate in social activities [1,2]. In advanced animal societies, the genetic diversity resulting from sexual reproduction is thought to provide multiple benefits, including enhanced disease resistance, and resilient division of labor [3,4]. Termite colonies commonly comprise both male and female reproductives, workers, and soldiers, which often exhibit sex-based phenotypic differences in the workforce [5,6]. The complete loss of males from termite lineages would therefore result not only in the loss of genetic diversity but also the loss of sex-based phenotypic diversity form their societies. We have recently reported asexual societies in a lineage of the termite *Glyptotermes nakajimai* (Isoptera: Kalotermitidae) – a dramatic transition from mixed-sex to all-female asexual societies. This finding provides evidence that males are not indispensable in advanced animal societies, regardless of whether males engage in social activities or not [7].

Some maternally inherited endosymbiotic bacteria, such as *Wolbachia, Cardinium*, and *Rickettsia*, can manipulate host reproduction in order to increase the number of infected hosts within a population: (*i*) by parthenogenesis induction (PI), in which infected females produce infected daughters without fertilization by males, (*ii*) by feminization of genetic males (FM), in which infected genetic males develop phenotypically as females, (*iii*) by male killing (MK), in which infected males are killed early in embryonic development, and (*iv*) cytoplasmic incompatibility (CI), in which uninfected females produce few or no offspring when they mate with infected males [8–10]. Here, we examine the possibility of endosymbiont-induced host manipulations, such as PI, FM, MK, and CI in *G. nakajimai*.

## Results and discussion

**Figure 1.**
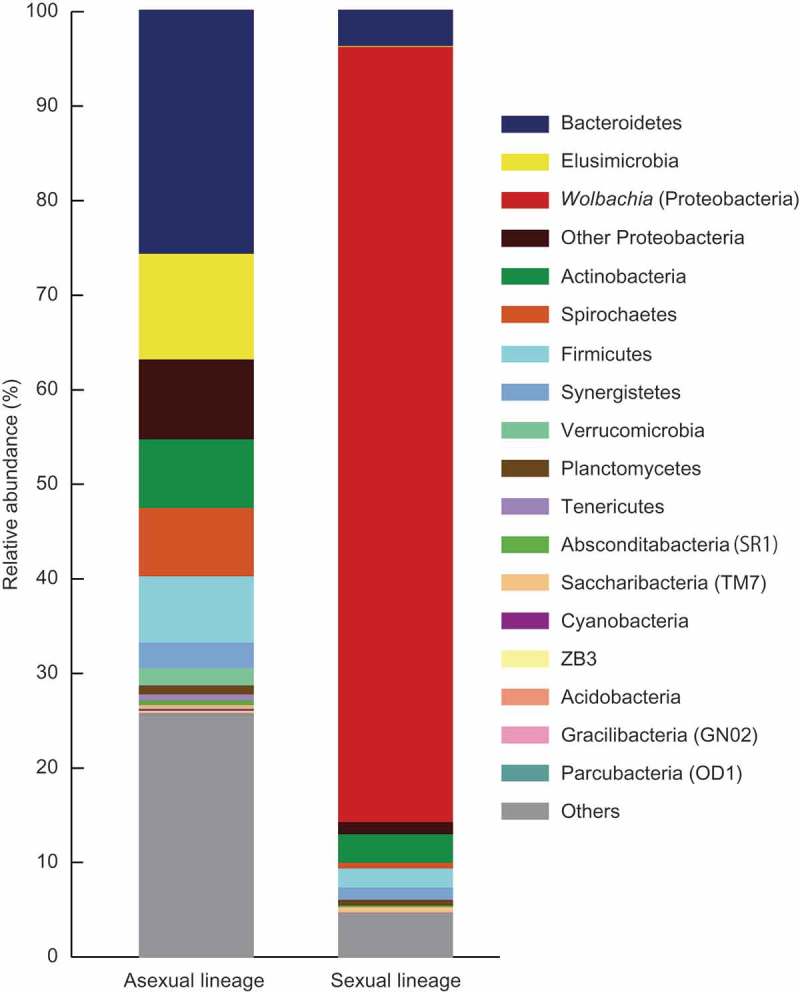
Bacterial taxa associated with the termite *Glyptotermes nakajimai*. The left bar graph is for the asexual lineage and the right is for the sexual lineage. Bacterial screening is based on whole workers (excluding guts) (*n* = 15).

We performed metabarcoding of the V1–V3 region of the 16S bacterial ribosomal RNA gene, amplified from DNA extracts of both asexual and sexual lineages of *G. nakajimai*. In total, 510 Operational Taxonomic Units (OTUs) and 318 OTUs were identified and assigned to multiple bacterial taxa for the asexual and sexual lineages, respectively (Figure 1). In the asexual lineage, bacteria that are commonly known to act as causal agents of parthenogenetic reproduction via PI (i.e., *Wolbachia, Cardinium*, and *Rickettsia*) as well as other endosymbiotic bacteria that are able to manipulate the reproduction of their hosts via FM and MK were not detected. In contrast, an abundance of *Wolbachia* (Supergroup F) was detected in the sexual lineage (Table 1 and Figure 1). This suggests that the asexuality in *G. nakajimai* is likely to be maintained without manipulations by endosymbiotic bacteria.

**Table 1.** Examination of bacterial endosymbionts associated with host reproductive manipulations in the asexual and sexual lineages of the termite *Glyptotermes nakajimai*. Bacterial screening is based on whole workers (excluding guts) (*n* = 15).

Assigned taxonomy		Relative abundance (%)	
(Phylum: Class: Genus)	Possible phenotype*	Asexual lineage	Sexual lineage
Proteobacteria: Alphaproteobacteria: *Rickettsia*	PI, MK	0	0
Proteobacteria: Alphaproteobacteria: *Wolbachia*	PI, FM, MK, CI	0	81.8
Proteobacteria: Alphaproteobacteria: Gen.^†^	CI^†^	0	0
Proteobacteria: Gammaproteobacteria: *Arsenophonus*	MK	0	0
Bacteroidetes: Cytophagia: *Cardinium*	PI, FM, CI	0	0
Bacteroidetes: Flavobacteriia: *Flavobacterium*	MK	0	0
Tenericutes: Mollicutes: *Spiroplasma*	MK	0	0

*Reviewed by Cordaux *et al*. [8]. PI, parthenogenesis induction; FM, feminization of genetic males; MK, male killing; CI, cytoplasmic incompatibility. ^†^Reported by Takano *et al*. [10].

Parthenogenesis induced by endosymbiotic bacteria has only been confirmed in host taxa with haplo-diploid sex determination (in which males develop from unfertilized haploid eggs and females from fertilized eggs), although the strong overrepresentation of haplo-diploids among species with known endosymbiont-induced parthenogenesis may be due to an ascertainment bias [9]. Termites are diploid and typically have an XY sex determination system [11]. Notably, four of six examined termite species with facultative parthenogenesis do not appear to harbour endosymbiotic bacteria [12,13]. These results suggest that endosymbiotic bacteria are unlikely to be the inducers of parthenogenesis in termites.

Lack of endosymbiotic bacteria in the asexual lineage of *G. nakajimai* (Table 1 and Figure 1) is suggestive of another selective force in the evolution of asexuality in the lineage. Indeed, Yashiro *et al*. [7] have demonstrated that asexual colonies of *G. nakajimai* have a more uniform head size in their all-female soldier caste and fewer soldiers in proportion to other individuals compared with sexual colonies, indicating increased defensive efficiencies arising from asexuality. However, we are unable to rule out the possibility that endosymbiont DNA that causes parthenogenesis was integrated into the host genome, followed by the loss of the donor endosymbiont [14,15]. Further work involving screening of candidate genes for parthenogenesis is required to investigate this possibility.

The asexual and sexual lineages of *G. nakajimai* are distributed allopatrically, with the asexual populations in Shikoku and Kyushu and the sexual populations in Honshu, Amami-Oshima Island, Okinawa Island, and Ogasawara Islands, Japan [16]. Dry-wood termites, including *G. nakajimai*, are single site nesters, living in a single piece of dead wood that serves both as nest and food [17], which facilitate human-mediated dispersal [18,19]. These dry-wood nesters can also easily disperse over water through wood rafting [20,21]. Therefore, males of *G. nakajimai* may migrate from sexual populations to asexual populations, potentially leading to sexualization of asexual populations if asexual females can still reproduce sexually. Our bacterial screening revealed a clear contrast between the asexual lineage without *Wolbachia* and the sexual lineage with *Wolbachia* (Table 1 and Figure 1), which might lead to CI between the asexual and sexual lineages. Further work is required to investigate the possibility that *Wolbachia*-induced CI contribute to the maintenance of asexuality in the lineage. Yashiro *et al*. [7] have illustrated that the two lineages containing different chromosome profiles (2n = 35 in the asexual lineage vs 2n = 34 in the sexual lineage), which might also act as a reproductive barrier between the two lineages.

## Methods

We extracted genomic DNA from two pools of samples. Pool 1 contained 15 female workers (also called pseudergates which predominantly develop into alates in the family Kalotermitidae [22]) (five each from three different colonies collected from Tokushima [Shikoku], Ashizuri [Shikoku], and Saiki [Kyushu], Japan, respectively) from the asexual lineage and Pool 2 contained 15 female workers (five each from three different colonies collected from Kushimoto [Honshu], Amami-Oshima Island, and Ogasawara Islands, Japan, respectively) from the sexual lineage of *G. nakajimai*. Prior to DNA extraction, samples were surface sterilized by washing in 10% bleach for ca. 30 s and rinsed in 95% ethanol to remove residual bleach before DNA extraction. DNA was extracted from the whole termites (excluding guts) using the DNeasy Blood & Tissue Kit (Qiagen, Valencia, CA, USA) following the manufacturer’s supplied protocol. Genomic DNA was sent to a commercial facility (the Australian Genome Research Facility [AGRF, www.agrf.org.au]) for Illumina MiSeq sequencing of the V1–V3 region of the 16S ribosomal RNA gene. The region V1–V3 was amplified with primers 27F/519R [23]. Sequencing reads were initially analyzed using the Quantitative Insights Into Microbial Ecology (QIIME) [24] and the USEARCH software [25,26]. After excluding all sequences with read length <240 bp and sequences with ambiguous base reads, 42,934 reads from Pool 1 and 68,113 reads from Pool 2 were included in the analysis. OTU tables were summarized at different taxonomic levels within QIIME using the Greengenes 13_8 database [27]. The 16S gene sequence of *Wolbachia* obtained in this study was deposited in the DDBJ/EMBL/GenBank nucleotide sequence databases under the accession number MK572633. BLAST search at the NCBI (http://www.ncbi.nlm.nih.gov) was used for identification of the *Wolbachia* supergroup.

## Abbreviations

PI: parthenogenesis inducing
FM: feminization of genetic males
MK: male killing
CI: cytoplasmic incompatibility
OUT: operational taxonomic unit

## References

[1] Bird R. Cooperation and conflict: the behavioral ecology of the sexual division of labor. Evol Anthropol. 1999;8:65–75.

[2] Wilson EO Sociobiology: the new synthesis, 25th anniversary edition. Cambridge, MA: Harvard University Press; 2000.

[3] Oldroyd BP, Fewell JH Genetic diversity promotes homeostasis in insect colonies. Trends Ecol Evol. 2007;22:408–413. 17573148 10.1016/j.tree.2007.06.001

[4] Ross-Gillespie A, O’Riain MJ, Keller LF Viral epizootic reveals inbreeding depression in a habitually inbreeding mammal. Evolution. 2007;61:2268–2273. 17767596 10.1111/j.1558-5646.2007.00177.x PMC7202238

[5] Matsuura K A novel hypothesis for the origin of the sexual division of labor in termites: which sex should be soldiers? Evol Ecol. 2006;20:565–574.

[6] Noirot C Social structure in termite societies. Ethol Ecol Evol. 1989;1:1–17.

[7] Yashiro T, Lo N, Kobayashi K, et al Loss of males from mixed-sex societies in termites. BMC Biol. 2018;16:96. 30249269 10.1186/s12915-018-0563-y PMC6154949

[8] Cordaux R, Bouchon D, Grève P The impact of endosymbionts on the evolution of host sex-determination mechanisms. Trends Genet. 2011;27:332–341. 21663992 10.1016/j.tig.2011.05.002

[9] Ma W-J, Schwander T Patterns and mechanisms in instances of endosymbiont-induced parthenogenesis. J Evol Biol. 2017;30:868–888. 28299861 10.1111/jeb.13069

[10] Takano S, Tuda M, Takasu K, et al Unique clade of alphaproteobacterial endosymbionts induces complete cytoplasmic incompatibility in the coconut beetle. Proc Natl Acad Sci USA. 2017;114:6110–6115. 28533374 10.1073/pnas.1618094114 PMC5468645

[11] Bergamaschi S, Dawes-Gromadzki TZ, Scali V, et al Karyology, mitochondrial DNA and the phylogeny of Australian termites. Chromosome Res. 2007;15:735–753. 17622491 10.1007/s10577-007-1158-6

[12] Hellemans S, Kaczmarek N, Marynowska M, et al Bacteriome-associated *Wolbachia* of the parthenogenetic termite *Cavitermes tuberosus*. FEMS Microbiol Ecol. 2019;95:fiy235. 10.1093/femsec/fiy235 30551145

[13] Matsuura K, Fujimoto M, Goka K Sexual and asexual colony foundation and the mechanism of facultative parthenogenesis in the termite *Reticulitermes speratus* (Isoptera, Rhinotermitidae). Insectes Soc. 2004;51:325–332.

[14] Hamilton PT, Hodson CN, Curtis CI, et al Genetics and genomics of an unusual selfish sex ratio distortion in an insect. Curr Biol. 2018;28:3864–3870. 30449670 10.1016/j.cub.2018.10.035

[15] van der Kooi CJ, Schwander T Evolution of asexuality via different mechanisms in grass thrips (Thysanoptera: aptinothrips). Evolution. 2014;68:1883–1893. 24627993 10.1111/evo.12402

[16] Takematsu Y, Yamaoka R Taxonomy of *Glyptotermes* (Isoptera, Kalotermitidae) in Japan with reference to cuticular hydrocarbon analysis as chemotaxonomic characters. Esakia. 1997;37:1–14.

[17] Abe T Evolution of life types in termites In: Kawano S, Connell JH, Hidaka T, editors. Evolution and coadaptation in biotic communities. Tokyo, Japan: University of Tokyo Press; 1987 p. 125–148.

[18] Evans TA, Forschler BT, Grace JK Biology of invasive termites: a worldwide review. Annu Rev Entomol. 2013;58:455–474. 23020620 10.1146/annurev-ento-120811-153554

[19] Yashiro T, Mitaka Y, Nozaki T, et al Chemical and molecular identification of the invasive termite *Zootermopsis nevadensis* (Isoptera: archotermopsidae) in Japan. Appl Entomol Zool. 2018;53:215–221.

[20] Emerson AE, Schmidt KP Geographical origins and dispersions of termite genera. Fieldiana Zool. 1955;37:465–521.

[21] Scheffrahn RH, Křeček J, Chase JA, et al Taxonomy, biogeography, and notes on termites (Isoptera: Kalotermitidae, Rhinotermitidae, Termitidae) of the Bahamas and Turks and Caicos Islands. Ann Entomol Soc Am. 2006;99:463–486.

[22] Korb J, Thorne B Sociality in termites In: Rubenstein DR, Abbot P, editors. Comparative social evolution. Cambridge, UK: Cambridge University Press; 2017 p. 84–123.

[23] Lane DJ 16S/23S rRNA sequencing In: Stackbrandt E, Goodfellow M, editors. Nucleic acid techniques in bacterial systematics. Cambridge, UK: John Wiley and Sons Ltd; 1991 p. 115–175.

[24] Caporaso JG, Kuczynski J, Stombaugh J, et al QIIME allows analysis of high-throughput community sequencing data. Nat Methods. 2010;7:335–336. 20383131 10.1038/nmeth.f.303 PMC3156573

[25] Edgar RC Search and clustering orders of magnitude faster than BLAST. Bioinformatics. 2010;26:2460–2461. 20709691 10.1093/bioinformatics/btq461

[26] Edgar RC, Haas BJ, Clemente JC, et al UCHIME improves sensitivity and speed of chimera detection. Bioinformatics. 2011;27:2194–2200. 21700674 10.1093/bioinformatics/btr381 PMC3150044

[27] DeSantis TZ, Hugenholtz P, Larsen N, et al Greengenes, a chimera-checked 16S rRNA gene database and workbench compatible with ARB. Appl Environ Microbiol. 2006;72:5069–5072. 16820507 10.1128/AEM.03006-05 PMC1489311

